# Public perception on face mask wearing during COVID-19 pandemic in Malaysia: A cross sectional study

**DOI:** 10.1371/journal.pone.0303031

**Published:** 2024-08-27

**Authors:** Muhammad Zubir Yusof, Nur Faza Zinnirah Fadzil, Nik Nur Niska Azlin Kamaruzi, Maya Syazana Syamimi Ayazi

**Affiliations:** 1 Department of Community Medicine, Kulliyyah of Medicine, International Islamic University Malaysia, Kuantan, Pahang, Malaysia; 2 IIUM Health, Safety and Environment (IHSEN), Kulliyyah of Medicine, International Islamic University Malaysia, Kuantan, Pahang, Malaysia; Atish Dipankar University of Science and Technology, BANGLADESH

## Abstract

**Introduction:**

Since the emergence of COVID-19, the Malaysian government has made wearing a face mask in public mandatory since August 1, 2020, as an effort by the government to control the transmission of COVID-19. However, Malaysians’ willingness to wear face masks in public is unknown.

**Objective:**

Thus, this study aimed to evaluate their perception of face mask wearing during COVID-19 and its contributing factors.

**Methodology:**

A total of 1024 respondents, aged ≥ 18 years, participated in this online cross-sectional survey from October 2021 to December 2021. The Face Mask Perception Scale (FMPS) was used to measure their perceptions.

**Results:**

Most of the respondents perceived wearing a face mask as uncomfortable. Our findings also revealed statistically significant differences and a small effect (f^2^ = 0.04) in which respondents who were concerned about being infected by the virus perceived face mask wearing appearance positively (B = - 0.09 units of log-transformed, 95% CI = - 0.15, - 0.04), whereas married respondents perceived it negatively (B = 0.07 units of log-transformed, 95% CI = 0.03, 0.09). There were no statistically significant differences in other domains of FMPS.

**Conclusion:**

In conclusion, discomfort was a major complaint. Marital status and fear of COVID-19 infection affected their perceptions. The public health implications of these findings highlight the importance of addressing discomfort and societal perceptions, particularly those influenced by factors such as marital status and COVID-19 experience, to promote widespread acceptance and consistent usage of face masks, which is crucial in mitigating the spread of COVID-19.

## Introduction

Severe acute respiratory syndrome coronavirus 2 (SARS-CoV-2) is a virus that causes COVID-19 and can spread quickly through droplets from the mouth or nose when breathing, coughing, sneezing, or speaking [1]. It first emerged from Wuhan, Hubei Province, China, in December 2019 and rapidly spread throughout the world, prompting the World Health Organization (WHO) to declare a pandemic on March 12^th^, 2020 [2]. The COVID-19 outbreak also had an impact on Malaysia, where the Ministry of Health reported 4,450,566 COVID-19 cases as of November 15, 2022, including 36,554 deaths and 4,885,228 cases of recoveries [3].

WHO recommended a few precautions for the public to follow in order to prevent the transmission of COVID-19, including wearing a face mask, avoiding crowded places, frequent hand hygiene, physical distance, and observing coughing and sneezing etiquette [4]. However, there was inconsistency in the recommendation of wearing a face mask in the community [5]. Initially, face mask wear was restricted to healthcare workers and those with flu-like symptoms during the first wave of COVID-19 cases [6, 7]. Later, once it was discovered that asymptomatic carriers may spread the virus, it was recommended that everyone wear a face mask in public [8]. Due to these reasons, Malaysia has made the wearing of a face mask required in public spaces beginning August 1^st^, 2020, in compliance with WHO guidelines. Those who fail to comply with the regulations may face fines of up to MYR 1000 (approximately USD 200) under the Preventive and Control of Infectious Disease Act 1988 [9].

Even though wearing a face mask in public places has become compulsory, there have been occasions where people have defied this new face mask mandate. The perceptions received from the face mask may have an impact on this health behaviour [10, 11]. According to a recent meta-analysis study, self-reported perceptions of face mask wearing in the community may differ across countries or regions during the pandemic [12]. For example, in comparison to other regions, communities in the West Pacific had a higher perception rate that the face mask could reduce COVID-19 virus transmission. In Canada, there has been significant ambiguity, inconsistency, and mistrust surrounding public health messages regarding face masks due to frequent changes in recommendations regarding their usage and a lack of information regarding their proper use, effectiveness, and benefits [13]. Moreover, they experienced feelings of embarrassment and guilt when wearing medical-grade masks in non-medical contexts, despite their prioritization for healthcare workers during the initial shortage of personal protective equipment.

Sociodemographics, socioeconomics, COVID-19 infection, and face mask use have been investigated as factors and correlates of face mask use in the general population. The important determinants of face mask use in relation to sociodemographic characteristics include older age, female gender, living in an urban area, higher education levels, and married individuals [14–18]. Previous research also found that socioeconomic characteristics were significantly associated with wearing a face mask, which was indirectly linked to moral beliefs [19, 20]. For example, the likelihood of wearing a mask was found to be substantially related to those in the high-income group, who engaged more in self-protecting behaviours [21]. In terms of COVID-19 infection status, it showed that those who were worried about getting COVID-19 infection and the need to protect others from COVID-19 infection were significant predictors of wearing the face mask in public [22]. In addition, a study in Japan reported that more than half of their study population wore masks if they had close contact with COVID-19 patients [23]. Past experience with virus infection may also contribute to the individual’s decision to wear a face mask in public [24]. The type of face mask used and the length of time it was worn may influence the public’s perception of face mask wearing [25]. Based on this empirical evidence, we hypothesized that,

- sociodemographic and socioeconomic are associated with face mask wearer perception based on six domains such as comfort, efficacy doubts, inconvenience, attention, appearance, and access.
- the COVID-19 infection status is associated with face mask wearer perception based on six domains such as comfort, efficacy doubts, inconvenience, attention, appearance, and access.
- the face mask practice is associated with face mask wearer perception based on six domains such as comfort, efficacy doubts, inconvenience, attention, appearance, and access.

With the emergence of a new variant of COVID-19 and more relaxed COVID-19 protocols in Malaysia, a surge in cases is almost inevitable, and the face mask-wearing mandate may be re-imposed on the general population. Prior to this, it was necessary to investigate the public’s perception of their face mask wearing because their perceptions may differ and be influenced by several factors, including sociodemographics, socioeconomics, COVID-19 infection, and face mask practice. By examining these factors, we can obtain a better understanding of their perceptions and be better equipped to prevent the spread of the COVID-19 virus. Thus, we aimed to measure public perception based on specific domains in Face Mask Perception Scale (FMPS) and identify the factors that may influence it in Malaysia, particularly during the COVID-19 pandemic.

## Materials and methods

### Study design

An online, cross-sectional study was conducted using Google Forms from October 2021 until December 2021. The target estimated sample size of 1000 was determined by the average attention domain score of 7.25 (standard deviation = 3.2) (15), with a 2.9% margin of error, a 95% confidence interval, and a 50% non-response rate. The invitation to participate was distributed via social media such as Facebook, Twitter, Instagram, WhatsApp, and LinkedIn. The self-administered questionnaire was disseminated to reach as many respondents as possible throughout Malaysia during the data collection period, relying on the public survey groups that were available. The survey was open to all Malaysians aged 18 or older who could comprehend either Malay or English.

### Study instrument

This survey’s questionnaire was divided into four sections. The first section asked about the background characteristics of the respondents, such as age, gender, race, level of education, marital status, employment status, household income, and living area status. The second section includes questions related to the COVID-19 status, travel history to the COVID-19 cluster, history of close contact with a COVID-19 patient and worries about getting infected with the COVID-19 virus. The next section consists of three questions related to face mask usage, such as type, duration of wearing, and awareness of face mask fitting. The last section used the validated FMPS to measure the public perception of face mask wearing during the COVID-19 pandemic [26].

There were four items for each of the six dimensions in FMPS that were used in our study. These dimensions were comfort, efficacy doubts, inconvenience, attention, appearance, and access. Items are scored on a 7-point Likert scale ranging from 1 (strongly disagree) to 7 (strongly agree). The FMPS had undergone a validation process and demonstrated excellent reliability (a Cronbach alpha of 0.91). For each dimension, the mean was calculated, and higher scores represent more negative face mask perceptions [26].

A forward-backward translation was done according to WHO guidelines in order to translate the FMPS items between English and Malay by an independent translator who was blind to the objective of the study [27]. The face validity of the translated questionnaire was established by conducting a pre-test with two experts in public health and 15 volunteers to determine if they could comprehend the questions. The participants were instructed to highlight and clarify any ambiguous words, either by writing them down on a copy of the pretested questionnaire or by notifying one of the researchers. The internal consistency of the questionnaire was also evaluated using test-retest reliability.

### Statistical analysis

Data were analyzed using IBM SPSS Statistics, version 23.0 (IBM Corp., Armonk, NY) [28]. Descriptive statistics were used to describe the respondents’ background characteristics, COVID-19 status, and responses to face-mask-related questions. All continuous data were tested for normal distribution and log-transformed where necessary. Only the appearance and accessibility dimensions of the FPMS were not normally distributed in this study. For further analysis, they were log-transformed and shown as a geometric mean (GM). The association between the respondents’ background characteristics, COVID-19 status, and face mask-related questions was also examined using simple linear regression. Later, multiple linear regressions were performed. In our study, a linear relationship between the contributory factors and the FMPS domain scores was assumed after there was no evidence of multicollinearity. We checked for this problem by obtaining the tolerance for each independent variable to assume a linear relationship. A tolerance value of more than 0.4 is considered acceptable. Linearity was also checked by examining the assumption of equal variance using a scatter plot between residual (x) and predicted values (y) [29]. A final model was obtained by applying the enter method. We used enter method or forced entry approach to force all independent variables into the model simultaneously [30]. This method relies on our decision based on previous literature for including the chosen variables. It was also decided that a significance level of 0.25 was used to screen for the selected variable to be included in the multivariable model to assess the independent variables values [31].

The p value was adjusted using Bonferroni correction to minimize the risk of Type I error [32]. The Bonferroni correction was applied to adjust for the effects of multiple hypotheses testing. The α level, which represents the probability of making a type I error, was set to 0.0033. This value was obtained by dividing 0.05 (type I error probability) by 15 (the number of analyses). To enhance the practical significance and understanding of the results, the effect sizes (measures of the strength of associations) were also assessed. Based on commonly used effect size measures, the multiple regression coefficients (R^2^) of approximately 0.02, 0.13, and 0.26 are considered small, medium, and large effects, respectively. These values correspond to Cohen’s *f*^2^ (R^2^/1-R^2^) of 0.02, 0.15, and 0.35 [33].

### Ethical consideration

This research was approved by the Kulliyyah of Medicine Research Committee, International Islamic University Malaysia (IIUM) Kuantan (IIUM/305/13/26/1). Participation in the study was entirely voluntary. The written consent of the participants was obtained by clicking "I agree" in the online form before proceeding with the online self-administered questionnaire.

## Results

A total of 1024 respondents participated in this study. Table 1 shows the background characteristics, COVID-19 status, and face mask practice. Respondents had a mean age of 29.7 (standard deviation, SD = 12.6) years. Out of 1024 respondents, the majority were women (73.2%), Malay (96.5%), had a high education (89.1%), were single or had ever married (65.2%), were unemployed, pensioners, or students (58.7%), and lived in an urban area (61.3%). A greater proportion of the respondents (48.4%) had a B40 household income (<USD 1031), then a M40 household income (USD 1031–2330) (35.7%), and finally a T20 household income group (>USD 2330) (15.8%). Most of the respondents had never been diagnosed with COVID-19 (92.7%), had never attended any areas associated with a known COVID-19 cluster (85.8%), and had no close contact with a COVID-19 patient (76.2%).

**Table 1:** Background characteristics, COVID-19 status, and face mask practice (N = 1024).

Characteristics	n	%
Age (years)	29. 7 (12.6) *	
Gender		
Male	274	26.8
Female	750	73.2
Race		
Non-Malay	36	3.5
Malay	988	96.5
Level of education		
Low	112	10.9
High	912	89.1
Marital status		
Single / Ever married	668	65.2
Married	356	34.8
Employment status		
Employed	423	41.3
Unemployed/Pensioner/Student	601	58.7
Household income group^†^		
B40	496	48.4
M40	366	35.7
T20	162	15.8
Living Area Status		
Rural	396	38.7
Urban	628	61.3
COVID-19 status		
Never been diagnosed	949	92.7
Had been diagnosed before	75	7.3
Ever attended any areas associated with known COVID-19 cluster		
No	879	85.8
Yes	145	14.2
Ever had any close contact with COVID-19 patient		
No	780	76.2
Yes	244	23.8
Concerned that you or a family member could get infected with COVID-19		
No	52	5.1
Yes	972	94.9
Type of face mask wearing		
Others	886	86.5
Respirator (N95 and/or K94) only	138	13.5
Duration of wearing face mask in public (hours)		
< 4	485	47.4
≥ 4	539	52.6
Awareness on Face Mask Need to be Fitted to the Face		
No	41	4
Yes	983	96

*Mean (Standard deviation (SD)); ^†^B40, M40, and T20, represent percentages of the country’s population of Bottom 40%, Middle 40%, and Top 20% respectively. B40: <MYR 4,850 (<USD 1031); M40: 4,850–10,959 (USD 1031–2330); T20: > 10,959 (>USD 2330) [34].

Most respondents (94.9%) were also concerned that they or a family member could get infected with COVID-19. A small number of the respondents (13.5%) wore tight-fitting respirators (N95 and/or K94) only. Half of respondents (52.6%) wore face masks for four hours or more in public. Almost all (96%) of those surveyed were aware that a face mask needed to be fitted to the face. Overall, the Malay version of the questionnaire was deemed clear, straightforward, and simple to understand by the participants (S1 Table). The Cronbach’s alpha coefficients were satisfactory for comfort (0.83), efficacy doubts (0.65), inconvenience (0.87), attention (0.92), appearance (0.95), and accessibility (0.75) (18) (S2 Table).

Table 2 illustrates the responses regarding face mask perception. The average comfort score was 4.17 (1.44). More than half of the respondents (>60%) reported discomfort wearing a face mask due to difficulty breathing and facial overheating; however, they disagreed that face masks become too hot when worn. Additionally, they expressed no doubts about the efficacy of wearing face masks, with the majority disagreeing that face masks merely provide a false sense of security (70.6%), are ineffective (87.4%), or are unsafe (70.8%). The average efficacy doubt score was 3.35 (0.85). The average scores for inconvenience, attention, and appearance were 2.34 (1.16), 2.68 (1.44), and 1.26 (1.2) respectively. The majority did not believe that wearing a face mask caused inconvenience (>75%), bothered them with attention (>58%), or affected their appearance (>91%). Accessibility to obtain face masks was not an issue among the respondents, with an average score of 1.19 (1.23). The majority knew where to purchase a face mask (97.4%), where to buy the appropriate face mask (86.3%), found it easy to obtain a face mask (96.7%), and did not consider face masks to be too expensive to purchase (73.4%).

**Table 2:** Face mask perception responses.

Domain / Question	n (%)							Mean score (SD)
	Strongly disagree	Disagree	Somewhat disagree	Neither agree or disagree	Somewhat agree	Agree	Strongly agree	
Comfort								4.17 (1.44)
Face masks disrupt my breathing.	64 (6.3)	166 (16.2)	72 (7)	37 (3.6)	442 (43.2)	183 (17.9)	60 (5.9)	
It is difficult to breathe when wearing a face mask.	60 (5.9)	185 (18.1)	94 (9.2)	22 (2.1)	409 (39.9)	200 (19.5)	54 (5.3)	
Face masks cause me to overheat.	53 (5.2)	211 (20.6)	82 (8)	58 (5.7)	340 (33.2)	196 (19.1)	84 (8.2)	
Face masks get too hot.	102 (9.4)	290 (28.3)	106 (10.4)	119 (11.6)	234 (22.9)	112 (10.9)	61 (6)	
Efficacy doubts								3.35 (0.85)
Face masks provide few health benefits.	20 (2)	12 (1.2)	12 (1.2)	62 (6.1)	59 (5.8)	430 (42)	429 (41.9)	
Face masks just provide a false sense of security.	273 (26.7)	379 (37)	71 (6.9)	143 (14)	73 (7.1)	55 (5.4)	30 (2.9)	
Face masks are ineffective.	400 (39.1)	418 (40.8)	77 (7.5)	77 (7.5)	26 (2.5)	13 (1.3)	13 (1.3)	
Face masks are unsafe because they force you to touch your face.	229 (22.4)	394 (38.5)	101 (9.9)	170 (16.6)	87 (8.5)	32 (3.1)	11 (1.1)	
Inconvenience								2.34 (1.16)
I do not like remembering to wear a face mask.	366 (35.7)	388 (37.9)	79 (7.7)	74 (7.2)	45 (4.4)	49 (4.8)	23 92.2)	
I forget to wear a face mask when going out.	458 (44.7)	372 (36.3)	70 (6.8)	26 (2.5)	65 (6.3)	24 (2.3)	9 (0.9)	
Wearing a face mask is too much of a hassle.	312 (30.5)	353 (34.5)	98 (9.6)	49 (4.8)	146 (14.3)	44 (4.3)	22 (2.1)	
It is hard to develop the habit of wearing a face mask.	325 (31.7)	371 (36.2)	106 (10.4)	55 (5.4)	106 (10.4)	47 (4.6)	14 (1.4)	
Attention								2.68 (1.44)
Face masks make people seem untrustworthy.	317 (31)	269 (26.3)	78 (7.6)	129 (12.6)	154 (15)	49 (4.8)	28 (2.7)	
Face masks make people look suspicious.	272 (26.6)	240 (23.4)	77 (7.5)	117 (11.4)	210 (20.5)	68 (6.6)	40 (3.9)	
Face masks make others uncomfortable.	346 (33.8)	340 (33.2)	64 (6.3)	144 (14.1)	75 (7.3)	33 (3.2)	22 (2.1)	
Face masks make other people feel uneasy.	359 (35.1)	349 (34.1)	72 (7)	140 (13.7)	70 (6.8)	21 (2.1)	13 (1.3)	
Appearance								1.26 (1.2) †
Face masks look dumb.	531 (51.9)	351 (34.3)	50 (4.9)	49 (4.8)	19 (1.9)	11 (1.1)	13 (1.3)	
Face masks look silly.	579 (56.5)	339 (33.1)	36 (3.5)	34 (3.3)	15 (1.5)	8 (0.8)	13 (1.3)	
Face masks are ugly.	572 (55.9)	330 (32.2)	35 (3.4)	37 (3.6)	21 (2.1)	18 (1.8)	11 (1.1)	
Face masks look weird.	572 (55.9)	327 (31.9)	37 (3.6)	37 (4)	27 (2.6)	11 (1.1)	9 (0.9)	
Accessibility								1.19 (1.23) †
I do not know where to buy a face mask.	739 (72.2)	238 (23.2)	20 (2)	11 (1.1)	7 (0.7)	7 (0.7)	2 (0.2)	
There is nowhere for me to buy the proper type of face mask.	485 (47.4)	329 (32.1)	70 (6.8)	47 (4.6)	49 (4.8)	25 (2.4)	19 (1.9)	
It is difficult to get a face mask.	655 (64)	297 (29)	38 (3.7)	17 (1.7)	9 (0.9)	2 (0.2)	6 (0.6)	
Face masks are too expensive.	325 (31.7)	323 (31.5)	104 (10.2)	82 (8)	124 (12.1)	46 (4.5)	20 (2)	

Note: †Geometric mean

Several independent factors potentially associated with facemask perception domains were examined in simple linear regression. These factors were organized into three categories: background characteristics, COVID-19 status, and face mask practices. Marital status demonstrated a significant association with comfort; married individuals reported feeling more comfortable wearing a facemask compared to their counterparts (B = -0.27, 95% CI = -0.45, -0.09). Employment status also exhibited a significant association with comfort; respondents classified as unemployed, pensioners, or students (B = 0.27, 95% CI = 0.09, 0.45) reported experiencing more discomfort compared to employed respondents (see Table 3).

**Table 3:** Factors associated with comfort, efficacy doubts and inconvenience.

Variables	Comfort					Efficacy doubts					Inconvenience				
	Simple linear regression					Simple linear regression					Simple linear regression				
	B	SE	p-value	95%CI		B	SE	p-value	95%CI		B	SE	p-value	95%CI	
Age (years)	7x10^-5^	0.004	0.98	-0.01	0.01	-0.001	0.002	0.49	-0.01	0.003	0.003	0.003	0.33	-0.003	0.01
Gender	0.09	0.1	0.39	-0.11	0.29	-0.02	0.06	0.81	-0.13	0.1	-0.06	0.08	0.45	-0.22	0.09
Ethnicity	0.24	0.24	0.33	-0.24	0.72	-0.07	0.14	0.61	-0.36	0.21	0.07	0.19	0.73	-0.32	0.45
Level of education (ref: Low)	-0.01	0.14	0.96	-0.29	0.28	0.21	0.09	0.35	-0.25	0.09	-0.2	0.12	0.08	-0.43	0.02
Marital status (ref: Single/ever married)	-0.27	0.09	0.004**	-0.45	-0.09	0.09	0.06	0.1	-0.02	0.19	0.14	0.08	0.06	-0.01	0.29
Employment status (ref: Employed)	0.27	0.09	0.003**	0.09	0.45	-0.05	0.05	0.36	-0.16	0.06	0.02	0.07	0.78	-0.12	0.17
Household income (ref: B40)															
M40	-0.17	0.1	0.09	-0.36	0.03	-0.07	0.06	0.21	-0.19	0.04	-0.07	-0.07	0.39	-0.23	0.09
T20	-0.24	0.13	0.07	-0.49	0.02	-0.07	0.08	0.36	-0.22	0.08	-0.13	-0.13	0.22	-0.33	0.08
Living area (ref: Rural)	-0.01	0.09	0.95	-0.19	0.18	0.03	0.06	0.59	-0.08	0.14	-0.05	0.07	0.5	-0.19	0.09
COVID-19 Status (ref: Never been diagnosed)	0.01	0.17	0.87	-0.33	0.04	-0.04	0.1	0.68	-0.24	0.16	0.06	0.14	0.65	-0.21	0.34
Ever attended any event or areas associated with known COVID-19 cluster (ref: No)	0.02	0.13	0.94	-0.23	0.27	-0.18	0.08	0.02*	-0.33	-0.03	-0.16	0.1	0.12	-0.37	0.04
Ever had any close contact with COVID-19 patient before (ref: No)	0.05	0.11	0.62	-0.16	0.26	-0.002	0.06	0.97	-0.13	0.12	-0.02	0.09	0.81	-0.19	0.15
Are you concerned that you or a family member could get infected with COVID-19? (ref: No)	-0.21	0.2	0.31	-0.61	0.19	-0.15	0.12	0.23	-0.39	0.09	-0.41	0.16	0.01*	-0.74	-0.09
Type of face mask wearing (ref: Others)	-0.12	0.13	0.36	-0.38	0.14	-0.04	0.08	0.57	-0.11	0.19	-0.09	0.11	0.37	-0.3	0.11
Duration of wearing face mask in Public (ref: < 4 hours)	-0.06	0.09	0.54	-0.23	0.12	-0.13	0.05	0.01*	-0.24	-0.03	-0.16	0.07	0.03*	-0.29	-0.01
Awareness on face mask need to be fitted to the face (ref: No)	-0.16	0.23	0.49	-0.6	0.29	-0.17	0.14	0.22	-0.43	0.09	-0.47	0.18	0.01*	-0.84	-0.11

Note: Unstandardized coefficient (B), Standard error (SE), Confidence interval (CI); *p<0.05, **p<0.01, ***p<0.003 (Bonferroni adjusted)

It also revealed that those who had visited the COVID-19 cluster had no doubts about the mask’s efficacy (B = -0.18, 95% CI = -0.33, -0.03) compared to those who had never visited the affected area. Those wearing the facemask in public for four hours or more indicated that they were confident in the facemask’s efficacy (B = -0.13, 95% CI = -0.24, -0.03). It also appears that those who were concerned about protecting their families and themselves from the infection found it convenient to wear the facemask in public (B = -0.41, 95% CI = -0.74, -0.09). Those who wore a face mask for at least four hours (B = -0.16, 95% CI = -0.09, -0.21) and were aware that facemasks needed to be fitted (B = -0.47, 95% CI = -0.84, -0.11) perceived that wearing facemasks was convenient (Table 3).

The results presented in Table 4 show that a higher education level (B = -0.43, 95% CI = -0.71, -0.15) and belonging to the middle-income group (M40) (B = -0.21, 95% CI = -0.41, -0.02) were independently associated with the attention domain. Furthermore, individuals in the highest income group (T20) reported no difficulty in obtaining facemask supplies (B = -0.04 log-transformed units, 95% CI = -0.08, -0.01), whereas those who were aware that facemasks needed to be properly fitted reported easy access to facemask supplies (B = -0.09 units of log-transformed, 95% CI = -0.15, -0.03).

**Table 4:** Factors associated with attention and accessibility.

Variables	Attention					Accessibility				
	Simple linear regression					Simple linear regression				
	B	SE	p-value	95% CI		B	SE	p-value	95% CI	
Age (years)	3 x 10^−4^	0.004	0.93	-0.01	0.01	-0.001	4x10^-4^	0.17	-0.002	3x10^-4^
Gender	-0.01	0.1	0.89	-0.21	0.19	-0.01	0.01	0.34	-0.04	0.01
Ethnicity	-0.2	0.24	0.41	-0.68	0.28	-0.03	0.03	0.28	-0.09	0.03
Level of education (ref: Low)	-0.43	0.14	0.003**	-0.71	-0.15	-0.04	0.02	0.05	-0.07	0.001
Marital status (ref: Single/ever married)	0.1	0.09	0.28	-0.08	0.29	-0.002	0.01	0.85	-0.03	0.02
Employment status (ref: Employed)	0.04	0.09	0.68	-0.14	0.22	-0.003	0.01	0.82	-0.03	0.02
Household income (ref: B40)										
** **M40	-0.21	0.1	0.03*	-0.41	-0.02	-0.01	0.01	0.27	-0.04	0.01
** **T20	-0.23	0.13	0.07	-0.49	0.02	-0.04	0.02	0.01*	-0.08	-0.01
Living area (ref: Rural)	-0.02	0.09	0.8	-0.2	0.16	-0.01	0.01	0.56	-0.03	0.02
COVID-19 Status (ref: Never been diagnosed)	0.15	0.17	0.39	-0.19	0.49	0.04	0.02	0.07	-0.003	0.08
Ever attended any event or areas associated with known COVID-19 cluster (ref: No)	-0.17	0.13	0.18	-0.43	0.08	-0.02	0.02	0.31	-0.05	0.02
Ever had any close contact with COVID-19 patient before (ref: No)	-0.03	0.11	0.75	-0.24	0.17	0.01	0.01	0.3	-0.01	0.04
Are you concerned that you or a family member could get infected with COVID-19? (ref: No)	-0.27	0.2	0.19	-0.67	0.13	-0.02	0.03	0.57	-0.07	0.04
Type of face mask wearing (ref: Others)	-0.11	0.13	0.39	-0.37	0.15	-0.01	0.02	0.68	-0.04	0.03
Duration of wearing face mask in Public (ref: < 4 hours)	-0.12	0.09	0.18	-0.29	0.06	-0.02	0.01	0.09	-0.04	0.003
Awareness on face mask need to be fitted to the face (ref: No)	-0.31	0.23	0.18	-0.76	0.14	-0.09	4x10^-4^	0.002***	-0.15	-0.03

Note: Unstandardized coefficient (B), Standard error (SE), Confidence interval (CI); *p<0.05, **p<0.01, ***p<0.003 (Bonferroni adjusted)

Table 5 demonstrates that individuals with a low level of education (B = -0.08 units of log-transformed, 95% CI = -0.12, -0.03) and those who are married (B = 0.06 units of log-transformed, 95% CI = 0.03, 0.08) expressed concerns about their appearance while wearing a face mask. However, individuals who were cautious about protecting their families and themselves from infection did not perceive any issues with the appearance (B = -0.08 units of log-transformed, 95% CI = -0.14, -0.03). Additionally, those who wore a face mask for four hours or longer in public were not concerned about the appearance created by the face mask (B = -0.03 units of log-transformed, 95% CI = -0.05, -0.001).

**Table 5:** Factors associated with appearance.

	Appearance									
Variables	Simple linear regression					Multiple linear regression^†^				
	B	SE	p-value	95% CI		B	SE	p-value	95% CI	
Age (years)	4x10^-4^	0.001	0.44	-0.001	0.001	-	-	-	-	-
Gender	- 0.02	0.02	0.27	-0.05	0.01	-	-	-	-	-
Ethnicity	- 0.02	0.04	0.59	-0.09	0.05	-	-	-	-	-
Level of education (ref: Low)	-0.08	0.02	<0.003***	-0.12	- 0.03	-	-	-	-	-
Marital status (ref: Single/Ever married)	0.06	0.01	<0.003***	0.03	0.08	0.07	0.02	<0.003***	0.03	0.09
Employment status (ref: Employed)	-0.02	0.01	0.14	-0.05	0.01	-	-	-	-	-
Household income (ref: B40)								-		
** **M40	-0.01	0.01	0.62	-0.04	0.02	-	-	-	-	-
** **T20	-0.02	0.02	0.24	-0.06	0.02	-	-	-	-	-
Living area (ref: Rural)	-0.01	0.01	0.57	-0.03	0.02	-	-	-	-	-
COVID-19 Status (ref: Never been diagnosed)	0.02	0.03	0.49	-0.03	0.07	-	-	-	-	-
Ever attended any event or areas associated with known COVID-19 cluster (ref: No)	-0.03	0.02	0.09	-0.07	0.01	-	-	-	-	-
Ever had any close contact with COVID-19 patient before (ref: No)	-0.02	0.02	0.18	-0.05	0.01	-	-	-	-	-
Are you concerned that you or a family member could get infected with COVID-19? (ref: No)	-0.08	0.03	0.005**	-0.14	-0.03	-0.09	0.03	0.001***	-0.15	-0.04
Type of Face Mask Wearing (ref: Others)	-0.02	0.02	0.37	-0.05	0.02	-	-	-	-	-
Duration of Wearing Face Mask in Public (ref: < 4 hours)	-0.03	0.01	0.03*	-0.05	-0.001	-	-	-	-	-
Awareness on Face Mask Need to be Fitted to the Face (ref: No)	-0.03	0.03	0.34	-0.09	0.03	-	-	-	-	-

Note: Unstandardized coefficient (B), Standard error (SE), Confidence interval (CI)

*p<0.05

**p<0.01

***p<0.003 (Bonferroni adjusted); ^†^adjusted R^2^ = 0.038, effect size, *f* ^2^ = 0.04 (small effect)

After adjusting for the multiple linear regression model, all variables were no longer significant for the comfort, efficacy doubts, inconvenience, attention, and accessibility domains (S3–S5 Tables). However, the results with Bonferroni correction were consistent in appearance domain (Table 5). The only statistically significant difference and small effect (f^2^ = 0.04) were observed for those who are married (B = 0.07 units of log-transformed, 95% CI = 0.03, 0.09) and concerned about the safety of their family from getting infected (B = - 0.09 units of log-transformed, 95% CI = - 0.15, - 0.04). Both associated factors explained 4.7% of the variance in the case of facemask appearance. The married respondents were reported to be reluctant to wear the facemask due to the appearance it created compared to their counterparts. In contrast, those who worried about their family being infected by the virus had no issue with the appearance created by wearing a facemask.

## Discussion

Before the emergence of COVID-19, the use of face masks was not common in Malaysian society. As COVID-19 imposed serious threats, wearing a face mask as a preventive measure played a key role in reducing and controlling the infection. Our findings indicate that the respondents felt uncomfortable when wearing facemasks. As Malaysia is a hot and humid country, wearing a face mask may cause irritation due to sweating, restricted breathing, and the urge to remove it. Several previous studies corroborate our findings. For instance, if the mask was worn for an extended period of time, the increased pressure on the face may create heat, skin irritation, or even pressure marks [35–37]. This was mostly because of the confined space behind the mask, which allows nasal air to warm the sides of the cheeks and aggravate the sensations of heat and humidity [38, 39]. Our respondents, on the other hand, indicated that the facemask did not get excessively hot while they were wearing it. These claims could be attributed to the type of facemask material used. When the breathing resistance of a facemask is high, its surface temperature is low [39]. The heat transfer from the skin to the environment through the mask should be controlled to achieve an equilibrium of heat loss. It implies that masks that quickly remove heat and moisture from the face are anticipated to be more comfortable to use [40]. In addition, we discovered that marital and work status may influence the comfort of wearing a facemask, but they were no longer significant in multivariable analysis. In agreement with previous studies, marital and work status were not identified as determinants of facemask comfort [41, 42].

Our respondents expressed no doubts regarding the efficacy of face masks, except for the belief that they would provide few health benefits. Most of them refuted the claim that face masks create a false sense of security and found them to be effective and non-harmful. One possible explanation for this mixed response was that wearing face masks in public was mandatory during the pandemic and strictly enforced by health authorities. Furthermore, the inconsistency of guidelines on the necessity of wearing face masks from global health organizations may create confusion among the community [5, 8, 43]. They may be overwhelmed with information from numerous public health information sources, making it difficult to justify the recommended measures of facemask use [44, 45]. Therefore, the news and health information provided by health authorities should be succinct and easy to comprehend for all individuals. They must keep the public informed about the facts and benefits of face mask use. It is critical because facts and deceptions provided to individuals can influence their judgments of efficacy [26]. Although previous studies have shown that individuals who are confident in consistently wearing face masks in public, even in COVID-19 cluster areas, can reduce the transmission of COVID-19 infections [46, 47], our study reported no significant results in multivariable analysis.

Despite several of our respondents indicating that wearing a facemask could be uncomfortable, most of them claimed that it was convenient to wear. This indicates that they had no trouble developing the habit of wearing face masks. The findings may possibly be related to public social norms to use facemasks during the pandemic, as facemask wearing was positively influenced by social norms [48, 49]. Likewise, prior research has demonstrated that respondents who were particularly concerned about their family members getting infected during the pandemic were more inclined to accept face masks as a societal norm [50, 51]. A desire to shield themselves and their family members from COVID-19 may have impacted their willingness [52]. This also reflected their views on the convenience of wearing the facemask for more than four hours and being aware that the facemask should be fitted well when worn. Consequently, it implies that individuals preferred to wear a facemask with a good fit for virus transmission protection [53, 54]. However, our findings in multivariable analysis are inconsistent with these previous findings.

Additionally, our respondents perceived that wearing a face mask did not attract negative social attention, suggesting that it had no influence on visual attention or perceived trustworthiness [55, 56]. Conversely, when queried about the accessibility of face masks, most respondents reported no difficulty in purchasing them. This indicated that face masks were readily available and accessible due to initiatives implemented by the Malaysian government during the pandemic, such as setting a maximum retail price of Malaysian Ringgit (MYR) 1 (USD 0.20) per unit for disposable three-ply face masks on August 15, 2020 [57] and increasing production and supply [58]. This price setting and guarantee of demand were taken to ensure the public incurs a lower burden of out-of-pocket expenses for facemask compliance during the pandemic. The multivariable analysis did not show any significant results, but our bivariate analysis did show that people in the high-income group (T20) who knew how important it was to get a facemask fitted, had a significant relationship with how easy they thought it was to get a facemask on.

Previous research findings have reported that in low- and middle-income countries, the underprivileged group often faces challenges in affording face masks due to financial constraints [59–61]. Conversely, the high-income group is more likely to purchase face masks without hesitation, which can be attributed to their stable financial resources and perceived personal behavioral control [62]. In addition, individuals who engage in protective behaviors and are aware of the risks associated with COVID-19 are more likely to have easy access to face masks [32].

Lastly, we observed that our respondents thought the face mask was appealing. This may be observed among those concerned about infecting themselves and their families with COVID-19, who did not allow the mask’s appearance to deter them from wearing it. They may consider the rationale for wearing a face mask to be more important than their feelings or appearance [57]. This demonstrates that appearance was not the primary reason for avoiding public face mask use. However, married respondents in our study were more concerned with their appearance when wearing the facemask in public. There was a common belief that married individuals might not care about their appearance because they had more responsibilities and priorities in their lives than their physical appearance [58]. Other studies suggest that facial attractiveness is an important indicator of marital behavior, with both spouses behaving more favorably in relationships where wives are more appealing than their husbands and more unfavorably in relationships where husbands are more attractive than their wives [58]. Additionally, previous research has shown that wearing a facemask may diminish perceived attractiveness when only the upper face is visible [59, 63].

To our knowledge, this is one of the few studies undertaken on the public’s perception of face mask use in Malaysia’s general population. The use of an online survey during the COVID-19 pandemic that relied more on the internet gave our study an advantage in achieving a large sample size. However, sharing the survey link through the online platform might limit accessibility among those with no internet connection; as a result, our findings might not be generalizable. Our study had the advantage of exploring the perception of facemask wearing in public, which was mandatory during the pandemic in Malaysia. However, social desirability might also be introduced in our study because they might intentionally try to omit their own violation of social norms. Besides, self-reported responses in our survey might lead to reporting and recall bias.

The non-random sampling approach used in our study may also have potentially resulted in self-selection bias. There is a possibility that the random errors will be greater than those resulting from analysing the sample as a simple random sample. In addition, interactions across associated factors make it difficult to interpret values from multivariable regression. It is important to consider factors identified in this study only as factors associated with perceptions of face mask wearing during COVID-19 rather than causal factors. The results should be evaluated with caution to avoid the Table 2 fallacy, which implies that all contributory factor estimates can be interpreted similarly [60].

## Conclusion

In general, our findings indicate that most respondents found wearing a face mask to be uncomfortable. Our study also suggests that marriage influences the appearance of wearing a face mask. The COVID-19 experience also played a vital role in determining face mask wearing perception, especially on the appearance created from wearing the facemask. Consequently, when using the facemask mandate as an implementation prevention strategy, we would recommend health authorities and policymakers consider the significant determinants of background characteristics and COVID-19 status identified in our study when establishing a new evidence-based policy.

Public health authorities should focus on improving communication and education regarding the importance of wearing face masks, particularly addressing concerns about discomfort. Strategies could include providing information on different types of masks, proper fit and adjustment techniques, and the importance of mask-wearing for protecting oneself and others from COVID-19. Understanding that marital status influences perceptions of wearing face masks suggests that interventions may need to be tailored to specific demographic groups. For example, educational campaigns could be designed to target married individuals, addressing any unique concerns or barriers they may face in adopting mask-wearing behaviors.

Given that perceptions of appearance play a role in mask-wearing behavior, public health messaging should emphasize the importance of viewing mask-wearing as a responsible and socially beneficial behavior rather than a hindrance to appearance. Highlighting the role of masks in preventing the spread of COVID-19 and protecting vulnerable populations may help shift perceptions and reduce stigma associated with mask-wearing. With the possibility of a new COVID-19 wave in the future, the potential benefits of facemask wearing in the community to reduce the risk of COVID-19 transmission should be emphasized as an adjunct to other preventive measures such as social distancing, isolation, hand hygiene, and vaccination, but not as a replacement for other public health measures. Future work could also explore the effectiveness of risk communication factors in influencing the perception of the face mask.

## Supporting information

S1 Table Malay-translated Face Mask Perception Scale (Malay-FPMS). (PDF)

S2 Table Item-total correlations and Cronbach’s α coefficients for Malay-FPMS. (PDF)

S3 Table Multiple linear regression analysis for comfort and efficacy doubts. (PDF)

S4 Table Multiple linear regression analysis for inconvenience and attention. (PDF)

S5 Table Multiple linear regression analysis for accessibility. (PDF)

S1 Raw data (XLSX)
