# Supplementary material for: Public perception on face mask wearing during COVID-19 pandemic in Malaysia: A cross sectional study
Source: PLoS One. 2024 Aug 27;19(8):e0303031. doi: 10.1371/journal.pone.0303031 (PMC11349217; doi:10.1371/journal.pone.0303031)
Supplement: S1 Table — (PDF) [file pone.0303031.s001.pdf]

S1 Table Malay-translated Face Mask Perception Scale

Skala Persepsi Pelitup Muka

Arahan:

Sila nyatakan sejauh mana anda setuju atau tidak setuju dengan kenyatan-kenyataan berikut tentang pelitup muka, iaitu penutup muka jenis kain yang dipakai untuk mencegah penyebaran penyakit. Setiap soalan berikut bermula dengan: Apabila saya tidak memakai pelitup muka di tempat awam, sebabnya adalah....

Domain	Item
Keselesaan	• Pelitup muka mengganggu pernafasan saya<br>• Susah untuk saya bernafas apabila memakai pelitup muka<br>• Saya berasa sangat panas apabila memakai pelitup muka<br>• Pelitup muka menjadi terlalu panas
Keraguan keberkesanan	• Pemakaian pelitup muka memberikan beberapa manfaat kesihatan<br>• Pelitup muka memberikan perasaan keselamatan yang palsu<br>• Pelitup muka tidak berkesan<br>• Pemakaian pelitup muka tidak selamat kerana terpaksa menyentuh muka semasa memakainya
Mudah didapati	• Saya tidak tahu di mana hendak membeli pelitup muka<br>• Saya tidak dapat mencari jenis pelitup muka yang sesuai.<br>• Sukar untuk mendapatkan pelitup muka<br>• Pelitup muka terlalu mahal
Menyusahkan	• Saya tidak suka bahawa saya perlu ingat untuk memakai pelitup muka<br>• Saya terlupa untuk memakai pelitup muka apabila keluar<br>• Pemakaian pelitup muka adalah terlalu merumitkan<br>• Ia adalah sukar untuk menanam tabiat pemakaian pelitup muka
Penampilan	• Pelitup muka kelihatan dungu<br>• Pelitup muka kelihatan mengarut<br>• Pelitup muka kelihatan hodoh<br>• Pelitup muka kelihatan pelik
Perhatian	• Pelitup muka membuatkan seseorang kelihatan tidak boleh dipercayai<br>• Pelitup muka membuatkan seseorang kelihatan mencurigakan<br>• Pelitup muka membuatkan orang lain tidak selesa<br>• Pelitup muka membuatkan orang lain merasa tidak selamat
