# Supplementary material for: Public perception on face mask wearing during COVID-19 pandemic in Malaysia: A cross sectional study
Source: PLoS One. 2024 Aug 27;19(8):e0303031. doi: 10.1371/journal.pone.0303031 (PMC11349217; doi:10.1371/journal.pone.0303031)
Supplement: S2 Table — (PDF) [file pone.0303031.s002.pdf]

S2 Table Item-total correlations and Cronbach's α coefficients for Malay-FPMS (N=15)

Domains	Items	Corrected item-total correlations	Cronbach's α
Comfort	Face masks disrupt my breathing.	0.53	0.83
	It is difficult to breathe when wearing a face mask.	0.76	
	Face masks cause me to overheat.	0.65	
	Face masks get too hot.	0.71	
Efficacy doubts	Face masks provide few health benefits.	0.21	0.65
	Face masks just provide a false sense of security.	0.6	
	Face masks are ineffective.	0.38	
	Face masks are unsafe because they force you to touch your face.	0.59	
Accessibility	I do not know where to buy a face mask.	0.51	0.75
	There is nowhere for me to buy the proper type of face mask.	0.73	
	It is difficult to get a face mask.	0.68	
	Face masks are too expensive.	0.56	
Inconvenience	I do not like remembering to wear a face mask.	0.61	0.87
	I forget to wear a face mask when going out.	0.75	
	Wearing a face mask is too much of a hassle.	0.83	
	It is hard to develop the habit of wearing a face mask.	0.86	
Appearance	Face masks look dumb.	0.94	0.95
	Face masks look silly.	0.93	
	Face masks are ugly.	0.96	
	Face masks look weird.	0.8	
Attention	Face masks make people seem untrustworthy.	0.87	0.92
	Face masks make people look suspicious.	0.8	
	Face masks make others uncomfortable.	0.93	
	Face masks make other people feel uneasy.	0.76	
