# Supplementary material for: Public perception on face mask wearing during COVID-19 pandemic in Malaysia: A cross sectional study
Source: PLoS One. 2024 Aug 27;19(8):e0303031. doi: 10.1371/journal.pone.0303031 (PMC11349217; doi:10.1371/journal.pone.0303031)
Supplement: S3 Table — (PDF) [file pone.0303031.s003.pdf]

S3 Table Multiple linear regression analysis for comfort and efficacy doubts

Variables	Comfort†					Efficacy doubts‡				
	B	SE	p-value	95% CI		B	SE	p-value	95% CI	
Age (years)	-	-	-	-	-	-	-	-	-	-
Gender	-	-	-	-	-	-	-	-	-	-
Ethnicity	-	-	-	-	-	-	-	-	-	-
Level of education (ref: Low)	-	-	-	-	-	-	-	-	-	-
Marital status (ref: Single/Ever married)	-0.13	0.12	0.26	-0.36	0.09	0.14	0.06	<0.02*	0.03	0.26
Employment status (ref: Employed)	0.17	0.11	0.14	-0.05	0.39	-	-	-	-	-
Household income (ref: B40)										
M40	-0.11	0.1	0.29	-0.31	0.1	-0.1	0.1	0.09	-0.22	0.02
T20	-0.15	0.13	0.25	-0.41	0.1	-0.12	0.08	0.13	-0.27	0.04
Living area (ref: Rural)	-	-	-	-	-	-	-	-	-	-
COVID-19 Status (ref: Never been diagnosed)	-	-	-	-	-	-	-	-	-	-
Ever attended any event or areas associated with known COVID-19 cluster (ref: No)	-	-	-	-	-	-0.17	0.08	0.03*	-0.32	-0.02
Ever had any close contact with COVID-19 patient before (ref: No)	-	-	-	-	-	-	-	-	-	-
Are you concerned that you or a family member could get infected with COVID-19? (ref: No)	-	-	-	-	-	-0.17	0.12	0.16	-0.41	0.07
Type of Face Mask Wearing (ref: Others)	-	-	-	-	-	-	-	-	-	-
Duration of Wearing Face Mask in Public (ref: < 4 hours)	-	-	-	-	-	-0.13	0.05	0.02*	-0.46	0.07
Awareness on Face Mask Need to be Fitted to the Face (ref: No)	-	-	-	-	-	-0.19	0.14	0.15	-0.27	0.04

Note: Unstandardized coefficient (B), Standard error (SE), Confidence interval (CI); *p<0.05, **p<0.01, ***p<0.003 (Bonferroni adjusted); †adjusted R^2=0.008, ‡adjusted R^2=0.01
