# Supplementary material for: Public perception on face mask wearing during COVID-19 pandemic in Malaysia: A cross sectional study
Source: PLoS One. 2024 Aug 27;19(8):e0303031. doi: 10.1371/journal.pone.0303031 (PMC11349217; doi:10.1371/journal.pone.0303031)
Supplement: S4 Table — (PDF) [file pone.0303031.s004.pdf]

S4 Table Multiple linear regression analysis for inconvenience and attention

**Variables**	**Inconvenience†**					**Attention‡**				
	**B**	**SE**	**p-value**	**95% CI**		**B**	**SE**	**p-value**	**95% CI**	
Age (years)	-	-	-	-	-	-	-	-	-	-
Gender	-	-	-	-	-	-	-	-	-	-
Ethnicity	-	-	-	-	-	-	-	-	-	-
Level of education (ref: Low)	-0.13	0.12	0.26	-0.36	0.1	-0.38	0.15	0.01*	-0.66	-0.09
Marital status (ref: Single/Ever married)	0.19	0.08	0.01*	0.04	0.35	-	-	-	-	-
Employment status (ref: Employed)	-	-	-	-	-	-	-	-	-	-
Household income (ref: B40)										
M40	-0.09	0.08	0.26	-0.25	0.07	-0.18	0.1	0.06	-0.38	0.01
T20	-0.18	0.11	0.1	-0.39	0.04	-0.19	0.13	0.14	-0.45	0.06
Living area (ref: Rural)	-	-	-	-	-	-	-	-	-	-
COVID-19 Status (ref: Never been diagnosed)	-	-	-	-	-	-	-	-	-	-
Ever attended any event or areas associated with known COVID-19 cluster (ref: No)	-0.14	0.1	0.19	-0.34	0.07	-0.14	0.13	0.29	-0.39	0.12
Ever had any close contact with COVID-19 patient before (ref: No)	-	-	-	-	-	-	-	-	-	-
Are you concerned that you or a family member could get infected with COVID-19? (ref: No)	-0.46	0.16	0.005**	-0.79	-0.14	-0.28	0.2	0.18	-0.68	0.13
Type of Face Mask Wearing (ref: Others)	-	-	-	-	-	-	-	-	-	-
Duration of Wearing Face Mask in Public (ref: < 4 hours)	-0.15	0.1	0.03*	-0.29	-0.01	-0.1	0.09	0.25	-0.28	0.07
Awareness on Face Mask Need to be Fitted to the Face (ref: No)	-0.52	0.18	0.004**	-0.88	-0.16	-0.31	0.23	0.17	-0.76	0.14

Note: Unstandardized coefficient (B), Standard error (SE), Confidence interval (CI); *p<0.05, **p<0.01, ***p<0.003 (Bonferroni adjusted); †adjusted R^2=0.021, ‡adjusted R^2=0.01
