# Supplementary material for: Public perception on face mask wearing during COVID-19 pandemic in Malaysia: A cross sectional study
Source: PLoS One. 2024 Aug 27;19(8):e0303031. doi: 10.1371/journal.pone.0303031 (PMC11349217; doi:10.1371/journal.pone.0303031)
Supplement: S5 Table — (PDF) [file pone.0303031.s005.pdf]

S5 Table Multiple linear regression analysis for accessibility

Variables	Accessibility†				
	**B**	**SE**	**p-value**	**95% CI**	
Age (years)	-0.001	$4.5x10^{-4}$	0.16	-0.002	0.0002
Gender	-	-	-	-	-
Ethnicity	-	-	-	-	-
Level of education (ref: Low)	-0.03	0.02	0.12	-0.07	0.008
Marital status (ref: Single/Ever married)	-	-	-	-	-
Employment status (ref: Employed)	-	-	-	-	-
Household income (ref: B40)					
M40	-0.01	0.01	0.46	-0.03	0.02
T20	-0.04	0.02	0.03*	-0.07	-0.003
Living area (ref: Rural)	-	-	-	-	-
COVID-19 Status (ref: Never been diagnosed)	0.04	0.02	0.09	-0.01	0.08
Ever attended any event or areas associated with known COVID-19 cluster (ref: No)	-	-	-	-	-
Ever had any close contact with COVID-19 patient before (ref: No)	-	-	-	-	-
Are you concerned that you or a family member could get infected with COVID-19? (ref: No)	-	-	-	-	-
Type of Face Mask Wearing (ref: Others)	-	-	-	-	-
Duration of Wearing Face Mask in Public (ref: < 4 hours)	-0.02	0.01	0.09	-0.04	0.003
Awareness on Face Mask Need to be Fitted to the Face (ref: No)	-0.09	0.03	0.003**	-0.14	-0.03

Note: Unstandardized coefficient (B), Standard error (SE), Confidence interval (CI); *p<0.05, **p<0.01, ***p<0.003 (Bonferroni adjusted); †adjusted R^2=0.018
